# Nicotinamide riboside kinases regulate skeletal muscle fiber-type specification and are rate-limiting for metabolic adaptations during regeneration

**DOI:** 10.3389/fcell.2022.1049653

**Published:** 2022-11-09

**Authors:** Tanja Sonntag, Sara Ancel, Sonia Karaz, Paulina Cichosz, Guillaume Jacot, Maria Pilar Giner, José Luis Sanchez-Garcia, Alice Pannérec, Sofia Moco, Vincenzo Sorrentino, Carles Cantó, Jérôme N. Feige

**Affiliations:** ^1^ Nestle Institute of Health Sciences, Lausanne, Switzerland; ^2^ EPFL School of Life Sciences, Ecole Polytechnique Fédérale de Lausanne, Lausanne, Switzerland; ^3^ Nestle Institute of Food Safety & Analytical Sciences, Lausanne, Switzerland

**Keywords:** skeletal muscle, NAD+, nicotinamide riboside, NRK, muscle stem cell (satellite cell), muscle regeneration, mitochondria, fiber type

## Abstract

Nicotinamide riboside kinases (NRKs) control the conversion of dietary Nicotinamide Riboside (NR) to NAD^+^, but little is known about their contribution to endogenous NAD^+^ turnover and muscle plasticity during skeletal muscle growth and remodeling. Using NRK1/2 double KO (NRKdKO) mice, we investigated the influence of NRKs on NAD^+^ metabolism and muscle homeostasis, and on the response to neurogenic muscle atrophy and regeneration following muscle injury. Muscles from NRKdKO animals have altered nicotinamide (NAM) salvage and a decrease in mitochondrial content. In single myonuclei RNAseq of skeletal muscle, NRK2 mRNA expression is restricted to type IIx muscle fibers, and perturbed NAD^+^ turnover and mitochondrial metabolism shifts the fiber type composition of NRKdKO muscle to fast glycolytic IIB fibers. NRKdKO does not influence muscle atrophy during denervation but alters muscle repair after myofiber injury. During regeneration, muscle stem cells (MuSCs) from NRKdKO animals hyper-proliferate but fail to differentiate. NRKdKO also alters the recovery of NAD^+^ during muscle regeneration as well as mitochondrial adaptations and extracellular matrix remodeling required for tissue repair. These metabolic perturbations result in a transient delay of muscle regeneration which normalizes during myofiber maturation at late stages of regeneration *via* over-compensation of anabolic IGF1-Akt signaling. Altogether, we demonstrate that NAD^+^ synthesis controls mitochondrial metabolism and fiber type composition *via* NRK1/2 and is rate-limiting for myogenic commitment and mitochondrial maturation during skeletal muscle repair.

## Introduction

Skeletal muscle is a very plastic tissue that responds to a multitude of stimuli such as exercise or injuries as well as disuse and disease. While the latter cause muscle wasting *via* the atrophy and contractile dysfunction of muscle fibers, exercise and injuries stimulate structural and metabolic adaptations in fibers and regenerative mechanisms that mobilize tissue-residing muscle stem cells (MuSCs) as well as other accessory cells (Bentzinger et al., 2013; Frontera and Ochala, 2015). Nicotinamide adenine dinucleotide (NAD^+^) plays a central role in muscle metabolism as a critical co-factor for glycolysis, mitochondrial tricarboxylic acid (TCA) cycle and oxidative phosphorylation (OXPHOS) and is therefore essential to enable efficient performance of cellular energy metabolism (Krebs and Veech, 1969). Moreover, NAD^+^ also serves as a co-substrate for NAD^+^-consuming enzymes implicated in calcium signaling, DNA repair, and post-transcriptional regulation of cellular energy metabolism and MuSC activation (Krebs and Veech, 1969; Fulco et al., 2003; Tang and Rando, 2014; Ryall et al., 2015). NAD^+^ levels are increased during physiological situations where energy is depleted such as fasting and calorie/glucose restriction (Fulco et al., 2008; Cantó et al., 2010) or exercise (Cantó et al., 2009; Cantó et al., 2010; Costford et al., 2010). In contrast, NAD^+^ levels decline during aging and muscle pathologies such as dystrophies or mitochondrial myopathies (Ryu et al., 2016; Migliavacca et al., 2019; Pirinen et al., 2020; Janssens et al., 2022).

Skeletal muscle NAD^+^ production relies on the salvage of intracellular NAD^+^ metabolites as well as dietary vitamin B_3_ precursors such as niacin, nicotinamide (NAM), nicotinamide mononucleotide (NMN) and nicotinamide riboside (NR) (Frederick et al., 2016; Ratajczak et al., 2016; Fletcher et al., 2017). Supplementation of exogenous NR increases tissue NAD^+^ content in rodents (Ryu et al., 2016; Zhang et al., 2016) and recent studies have described the beneficial effect of dietary NR supplementation on mitochondria, muscle aging and MuSC function (Cerutti et al., 2014; Khan et al., 2014; Ryu et al., 2016; Zhang et al., 2016). NAD^+^ synthesis from NR takes place in a two-step reaction: nicotinamide riboside kinases (NRKs) 1 and 2 phosphorylate NR to NMN, which is subsequently converted to NAD^+^ by NMN adenylyltransferases (NMNATs) (Bieganowski and Brenner, 2004; Trammell et al., 2016). This path is not only essential for the utilization of NR as NAD^+^ precursor, but also for NMN, as NMN needs to be converted to NR in order to enter the cell (Nikiforov et al., 2011; Ratajczak et al., 2016). NRK1 is ubiquitously expressed in all tissues, while NRK2 has been exclusively found in skeletal muscle and, to a lower degree, in heart (Li et al., 1999; Ratajczak et al., 2016; Fletcher et al., 2017). Expression of NRK1 and 2 (encoded by the *Nmrk1* and *Nmrk2* genes, respectively) is increased during primary mouse myoblast differentiation *in vitro*, with *Nmrk2* mRNA expression peaking during the time of myoblast fusion (Fletcher et al., 2017). A role of NRK2b in zebrafish muscle development was reported for the regulation of basal lamina assembly, laminin polymerization and paxillin concentration at the myotendinous junction (Goody et al., 2010). In contrast, mice lacking either one or both NRKs develop normally with no obvious muscle phenotype at baseline (Ratajczak et al., 2016; Fletcher et al., 2017; Deloux et al., 2018). However, the detailed architecture and ECM composition of NRK KO mice was not evaluated. Given the reported implication in muscle architecture as well as the observation that *Nmrk2* is a damage-inducible transcript in muscle and even in non-muscle tissues (i.e. during neuronal injury) (Sasaki et al., 2006; Lavery et al., 2008; Aguilar et al., 2015; Xu et al., 2015; Diguet et al., 2018), we hypothesized that NRKs may be important for muscle plasticity during atrophy and regeneration.

The aim of this study was to determine the role of NRK1/2 in healthy skeletal muscle as well as in scenarios of muscle atrophy and regeneration. Using NRK1/2 dKO mice, we demonstrate that NR is an endogenous NAD^+^ precursor in muscle and that loss of NRKs increases NAM salvage. We further show that NRK1/2 regulate muscle NAD^+^ and mitochondrial adaptations, stem cell activity and tissue remodeling during regeneration but do not influence muscle atrophy after denervation.

## Methods

### NRK1/2 dKO mice

Full-body NRK1/2 dKO (NRKdKO) mice were generated on a pure C57BL/6NTac background (Taconic Biosciences) (Ratajczak et al., 2016). Briefly, exons three to seven of the *Nmrk1* gene or exons two to seven of the *Nmrk2* gene were flanked with loxP sites. Floxed mice were crossed with mice expressing Cre recombinase under the general promoter of the Gt (ROSA)26Sor gene (Cre deleter). The deletion of these exons was validated by PCR and resulted in the complete loss of the NRK1 or NRK2 protein. Mice carrying the whole-body, including germ line, *Nmrk1* or *Nmrk2* deletion were further bred to eliminate expression of the Cre recombinase. NRKdKO mice were obtained by crossing single KO mice.

### *In vivo* experiments

All animal experiments were carried out according to Swiss and EU ethical guidelines and approved by the local animal experimentation committee of the Canton de Vaud under license number VD2764. Mice kept under a 12/12 h dark/light cycle and housed by three to five in ventilated cages with *ad libitum* access to water and food.

### Muscle regeneration

Skeletal muscle injuries were performed as previously described (Lukjanenko et al., 2019). Age- and body weight-matched animals were randomly assigned into different treatments. Both male and female mice were used. Mice were anesthetized with isoflurane inhalation prior to shaving one leg. Using a 22-gauge needle (Hamilton), *tibialis anterior* (TA) and *gastrocnemius* (GC) muscles were injected through one and two 50 µl injections of cardiotoxin (CTX, Latoxan, #L8102), diluted at 10 µM in physiological serum respectively. For muscle collection, mice were euthanized 7 and 14 days post-injury (dpi) with CO2 (air mixture 85:15) in an inhalation chamber followed by cervical dislocation. Both contralateral (CL) and injured muscles were dissected free of fat, weighted and snap frozen in liquid nitrogen. TA muscles were cut in two parts, one part was snap frozen in liquid nitrogen for total RNA and NAD^+^ extraction, the other part was embedded into optimal cutting temperature (OCT) matrix and then frozen for histological analyses in isopentane cooled with liquid nitrogen.

### Sciatic nerve resection

Mice were anesthetized with isoflurane inhalation prior to shaving one leg and performing an incision in the mid-thigh region. The sciatic nerve was isolated and a piece of approximately 5 mm was resected. The incision was closed with suture thread. Tissue collection was performed 7 days after surgery as described above.

### Immunohistochemistry

Frozen embedded TA muscle was sectioned to 10 µm on a cryostat and histology staining was performed as previously described (Dammone et al., 2018). For the Pax7, Ki67 and Myogenin staining, sections were dried for 10 min, then fixed for 10 min in 4% PFA, and permeabilized in 0.5% Triton X-100 (Sigma, X100) diluted in PBS (PBTX) for 10 min at room temperature (RT). For antigen retrieval, slides were immersed twice in hot citric acid (0.01 M, pH 6) placed in a boiling water bath for 5 min. Sections were subsequently blocked in 4% BSA (IgG-free Bovine Serum Albumin (BSA), Jackson #001-000-162) in PBS for 3h, followed by 30 min blocking with a goat-anti-mouse FAB (Jackson #115-007-003) at 1/50 in PBS. We next incubated the slides with primary antibodies anti-Pax7 (DSHB, purified, 2.5 μg/ml), anti-Myogenin (Abcam, #ab124800, 1/500), and anti-Ki67 (Thermo Fischer Scientific, #14-5698-82, 1/500). Pax7 signal was further amplified using a goat-anti mouse IgG1-biotin (Jackson ImmunoResearch #115-065-205, 1/1,000) followed by conjugation with Streptavidin AF555 (Life Technologies, #S-21381, 1/2000), and other antibodies were detected with their specific secondary antibodies (Thermo Fisher Scientific, #A32731; SouthernBiotech, #3065-31), while nuclei were detected with Hoechst 33342 (Sigma, #B2261). Slides were then mounted using Dako fluorescent mounting medium (Agilent, #S302380-2) and imaged with an Olympus VS120 slide scanner. Images were analyzed using the VS-ASW FL software measurement tool. The number of Pax7, Myogenin, and Ki67 positive cells in the injured region was determined by manually counting several random areas on entire muscle sections with researchers blinded to the experimental groups. A minimum of 300 Pax7+ cells were quantified from at least 0.5 mm^2^ of injured tissue for each mouse.

For laminin immunostaining, cryosections were blocked for 45 min at RT in 4% BSA. Cryosections were stained for 3 h at RT using anti-laminin antibody (Sigma, #L9393, 1/1,000). Secondary antibodies AF488-goat anti-rabbit (Thermo Fisher Scientific, #A11034) were incubated for 1 h in blocking solution and counterstained with Hoechst. Stained tissue sections were imaged using an Olympus VS120 slide scanner and analyzed using an automated image processing algorithm developed internally, followed by manual quality control. Cross sectional area (CSA) was detected by laminin/DAPI staining and measured for all regenerating myofibers with centralized nuclei of the section.

Finally, for the fiber typing staining, sections were subsequently blocked in 4% BSA (IgG-free Bovine Serum Albumin (BSA), Jackson #001-000-162) in PBS for 3h, followed by 30 min blocking with a goat-anti-mouse FAB (Jackson #115-007-003) at 1/50 in PBS. Slides were next incubated with anti-MHC 2B (DSHB, clone BFF3 purified, 1/10) and anti-laminin antibody (Sigma, #L9393, 1/1,000) overnight at 4 °C. Secondary antibodies AF555-goat anti-mouse (Thermo Fisher Scientific, #A21426) and AF405-goat anti-rabbit (Thermo Fisher Scientific, #A231556) were incubated for 1 h in blocking solution. Sections were then incubated with anti-MHC1 (DSHB, clone BAD5 purified, 1/400) and anti-MHC2A (DSHB, clone SC71 purified, 1/500) in blocking solution for 1h at RT and detected with AF488-goat anti-mouse (Thermo Fisher Scientific, #A21141) and AF647-goat anti-mouse (Thermo Fisher Scientific, #A21240) for another hour at RT. Stained tissue sections were imaged using an Olympus VS120 slide scanner and fiber type was analyzed across the entire sections on all fibers using an automated image processing algorithm developed internally using the QuPath software (Bankhead et al., 2017).

### Protein extraction and immunoblotting

Proteins from GC muscles of WT and NRKdKO animals were extracted in RIPA lysis and extraction buffer (Thermo Fisher Scientific, #89901) supplemented with protease (Sigma, #S8820) and phosphatase inhibitor cocktail (Sigma, #4906845001). Protein concentration was determined using a BCA assay (Thermo Fischer Scientific, #23227). Samples were first diluted to 1.5 mg/ml and boiled 5 min in NuPAGE™ LDS Sample Buffer (4X) (Invitrogen, #NP0007), run on 4–12% Bis-Tris Protein gels (Thermo Fischer Scientific, #BN1003), and transferred using the semi dry system from Life Technologies. Membranes were incubated overnight at 4°C with primary antibodies anti-NRK1 and anti-NRK2 (Ratajczak et al., 2016), anti-NAMPT from Bethyl, OxPhos antibody cocktail from Invitrogen, anti-VDAC, anti-TOM20, anti-IGF1R, anti-PI3K, anti-phospho-Akt (Ser473), anti-Akt, anti-Raptor, anti-FAK, and anti-Vinculin from Cell Signaling. Membranes were then washed and incubated for 1 h with horseradish peroxidase–conjugated donkey anti-rabbit (Jackson ImmunoResearch, #711-035-152, 1/5000). Proteins were visualized with chemiluminescent western blotting substrate (Thermo Fisher Scientific, #32132) using Amersham Hyperfilm™ films. Densitometry analysis was performed using Fiji. Protein levels in each lane were normalized to the levels of Vinculin as a loading control.

### Gene expression

Total mRNA from tissues was extracted with TRIzol (Life Technologies) and processed using the microRNA extraction kit (Qiagen) according to manufacturer’s instructions. mRNA was reverse-transcribed using the High Capacity cDNA Reverse Transcription Kit (Applied Biosystems) according to manufacturer’s protocol. Expression of mRNA was then measured by quantitative polymerase chain reaction (qPCR) using SYBR Green real time PCR technology and the Light Cycler 480 (Roche). Gene expression was normalized to housekeeping genes *Atp5b*, *Eif2a* and *Psmb4* for CTX experiments and to *Actinb* in the denervation study. Relative gene expression between groups or genotypes was assessed using the ΔΔCt method. The following primer sequences were used: *Atp5b*, forward: 5′-ACC​TCGGTG​CAG​GCT​ATC​TA-3′, reverse: 5′-AAT​AGC​CCG​GGA​CAA​CAC​AG-3’; *Eif2a*, forward: 5′-CAC​GGT​GCT​TCC​CAG​AGA​AT-3′, reverse: 5′-TGC​AGT​AGT​CCC​TTG​TTA​GCG-3’; *Psmb4*, forward: 5′-GCG​AGT​CAA​CGACAG​CAC​TA-3′, reverse: 5′-TCA​TCA​ATC​ACCATC​TGG​CCG-3’; *Nmrk1*, forward 5′-CCC​AAC​TGC​AGC​GTC​ATA​TC-3′, reverse: 5′-CCT​TGA​GCA​CTT​TCC​AAG​GC-3’; *Nmrk2*, forward: 5′-GAC​CAG​TCA​CCT​CCA​GTC​CC-3′, reverse: 5′-TTG​GTC​ACC​CCT​CCA​ATG​CC-3’; *Nmnat1*, forward: 5′-TGG​CTC​TTT​TAA​CCC​CAT​CAC-3′, reverse: 5′-TCT​TCT​TGT​ACG​CAT​CAC​CGA-3’; *Nnmt*, forward: 5′- AGC​TTT​GGG​TCC​AGA​CAC​TGT-3′, reverse: 5′-GAG​CCA​ATG​TCA​ATC​AGG​AGT​T-3’; *HDAC4*, forward: 5′-GCT​GAC​CTC​AGT​GTT​CGT​CA-3′, reverse: 5′-CTA​TCC​ACC​CCA​ACA​CCA​CC-3’; *TRIM63* (*MuRF1*), forward: 5′-GCT​ACC​TTC​CTC​TCA​AGT​GCC​A-3′, reverse: 5′-CAG​CCC​TTG​GAG​GCT​TCTACA-3’; *MAFbx* (atrogin1), forward: 5′-TGC​TCC​GTC​TCA​CTT​TCC​CC-3′, reverse: 5′-AGT​GTT​GTC-GTG​TGC​TGG​GA-3’; *Myogenin*, forward: 5′-GTG​CCC​AGT​GAA​TGC​AAC​TC-3’, reverse: 5′-CGCGAGCAAATGATCTCCTG-3′; *Ppgc1a*, forward: 5′-AAG​TGT​GGA​ACT​CTC​TGG​AAC​TG-3′, reverse: 5′-GGG​TTA​TCT​TGG​TTG​GCT​TTA​TG-3’; *Emr1* (F4/80), forward: 5′-CTC​TTC​TGG​GGC​TTC​AGT​GG-3′, reverse: 5′-TGT​CAG​TGC​AGG​TGG​CAT​AA-3’; *CD11b*, forward: 5′-GCC​TGT​GAA​GTA​CGC​CAT​CT-3′, reverse: 5′-GCC​CAG​GTT​GTT​GAA​CTG​GT-3’; *Msr1*, forward: 5′-ATT​GGC​TTC​CCT​GGA​GGT​CG-3′, reverse: 5′- GGA​GTT​ATA​CTG​ATC​TTGATC​CGC​C-3’; *TNFa*, forward: 5′-AGC​CGA​TGG​GTT​GTA​CCT​TG-3’; reverse: 5′- ATA​GCA​AAT​CGG​CTG​ACG​GT-3’; *IL-1b*, forward: 5′-TGC​CAC​CTT​TTG​ACA​GTG​ATG​A-3’; reverse: 5′-TGC​CTG​CCT​GAA​GCT​CTT​GT; *IL-6*, forward: 5-GGT​GAC​AAC​CAC​GGC​CTT​CCC-3′, reverse: 5′-AAG​CCT​CCG​ACT​TGT​GAA​GTG​GT-3′

### NAD^+^ measurements and LC-MS metabolomics

NAD^+^ levels were measured with an enzymatic cycling assay as previously described (Dall et al., 2018). Briefly, ca. 15 mg muscle tissue was lysed in 200 µL 0.6 M perchloric acid using a TissueLyser II (Qiagen). After centrifugation, the supernatant was diluted 500-fold in 100 mM Na_2_HPO_4_ pH 8.0.100 µL of diluted sample was combined with 100 µl reaction mix (100 mM Na_2_HPO_4_ pH 8, 2% ethanol, 90 U/ml alcohol dehydrogenase, 130 mU/ml diaphorase, 10 µM resazurin, 10 µM flavin mononucleotide, 10 mM nicotinamide), and the fluorescence increase at Ex/Em 540/580 nm was measured over 10 min. NAD^+^ content was calculated from a standard curve and normalized to tissue weight.

Sample preparation for LC-MS metabolomics analysis was based on Giner al (Giner et al., 2021). Samples (9- 18 mg muscle) were extracted with organic solvent, dried and reconstituted in 200 µl acetonitrile:water 60% (v/v). The analysis was performed by hydrophilic interaction chromatography (HILIC) ultra-high performance liquid chromatography mass spectrometry (UHPLC-MS), using a triple quadrupole MS TSQ Vantage (Thermo Fisher Scientific), (Giner et al., 2021). Positive ion mode extracted chromatograms of NR, NAM, *N* ^1^-methyl nicotinamide (MeNAM), and NAD^+^ were integrated and used for relative comparison. Retention time and mass detection was confirmed by authentic standards.

### Single-cell and single-nuclei RNA sequencing

The expression of NRK1, NRK2 and NAMPT was extracted from public datasets of single-cell RNAseq (mononucleated cells) and single nuclei (mononucleated and myofibers) RNAseq integrated in the scMuscle atlas by the Cosgrove lab (http://scmuscle.bme.cornell.edu/; (McKellar et al., 2021)) and in the MyoAtlas by the Millay lab (https://research.cchmc.org/myoatlas/; (Petrany et al., 2020)).

### Statistical analysis

GraphPad Prism Software version 9.02 for Windows was used for preparation of graphs and statistical analysis. Statistical methods were chosen as stated in the figure legends. Student’s t-test was used for comparison of two groups (WT vs NRKdKO) and one-way ANOVA was used to compare multiple groups. Kolmogorov-Smirnov test was used for comparison of fiber area distribution. All data are expressed as mean value ±s.e.m.

## Results

### Absence of NRK1 and NRK2 alters NAM salvage and causes fiber-type switching

**FIGURE 1:**
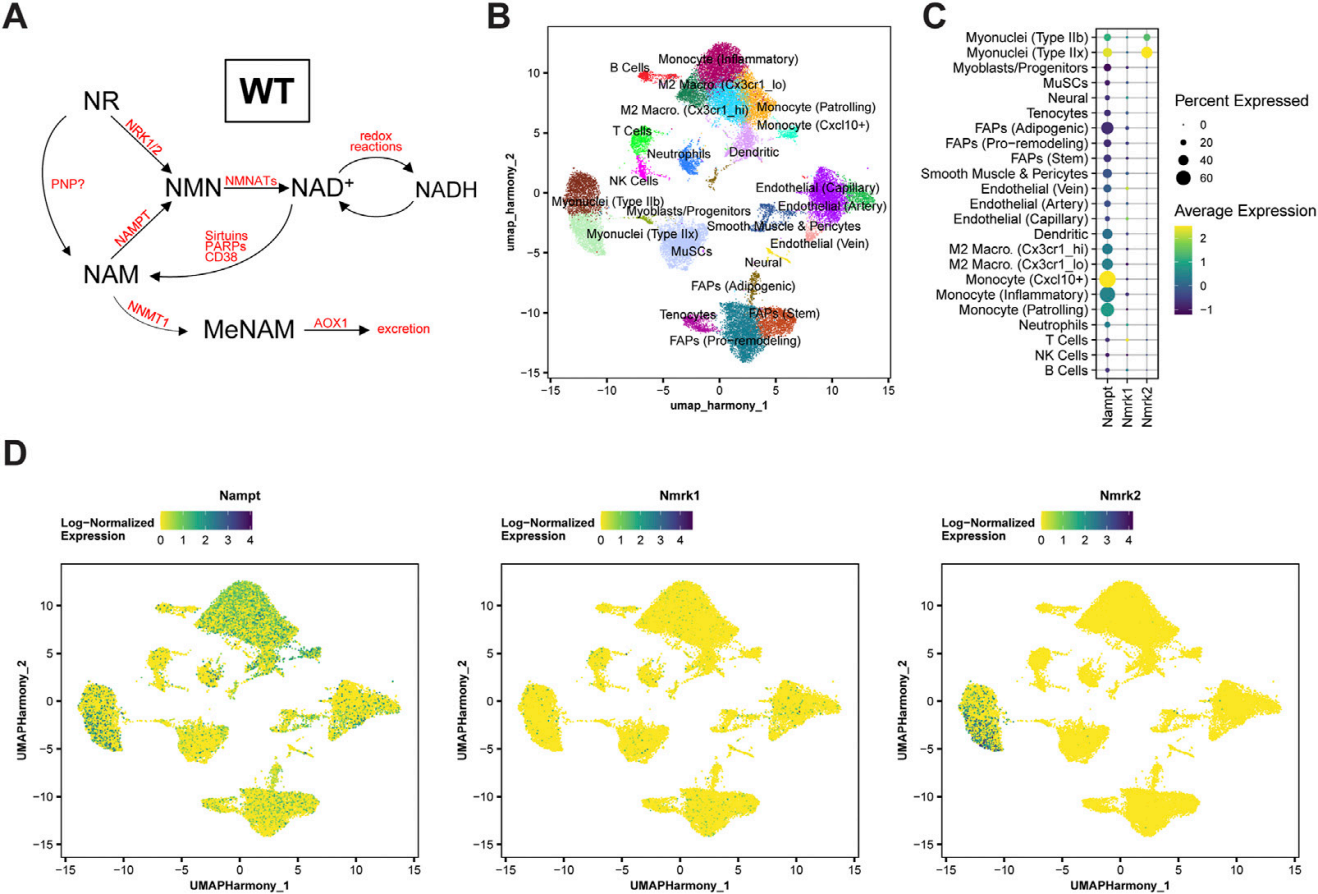
*Nampt*, *Nmrk1*, and *Nmrk2* expression dynamics in WT skeletal muscle. **(A)** Main NAD^+^ biosynthesis and consumption pathways in WT skeletal muscle. **(B–D)** Single-cell RNA-sequencing analysis of a notexin injury response in TA muscle of adult mice. TA muscle samples from 0, 1, 2, 3.5, 5, or 7 days post-injury (dpi) were analyzed from McKellar et al. (McKellar et al., 2021). **(B)** UMAP projection of scRNAseq data demonstrating cell-type annotations of clusters. **(C,D)** Dot plots **(C)** and UMAP projections **(D)** showing expression of *Nampt*, *Nmrk1*, and *Nmrk2* by cell-type cluster. Dot size shows the frequency of cells expressing non-zero transcript level. Dot color shows average expression level.

**FIGURE 2:**
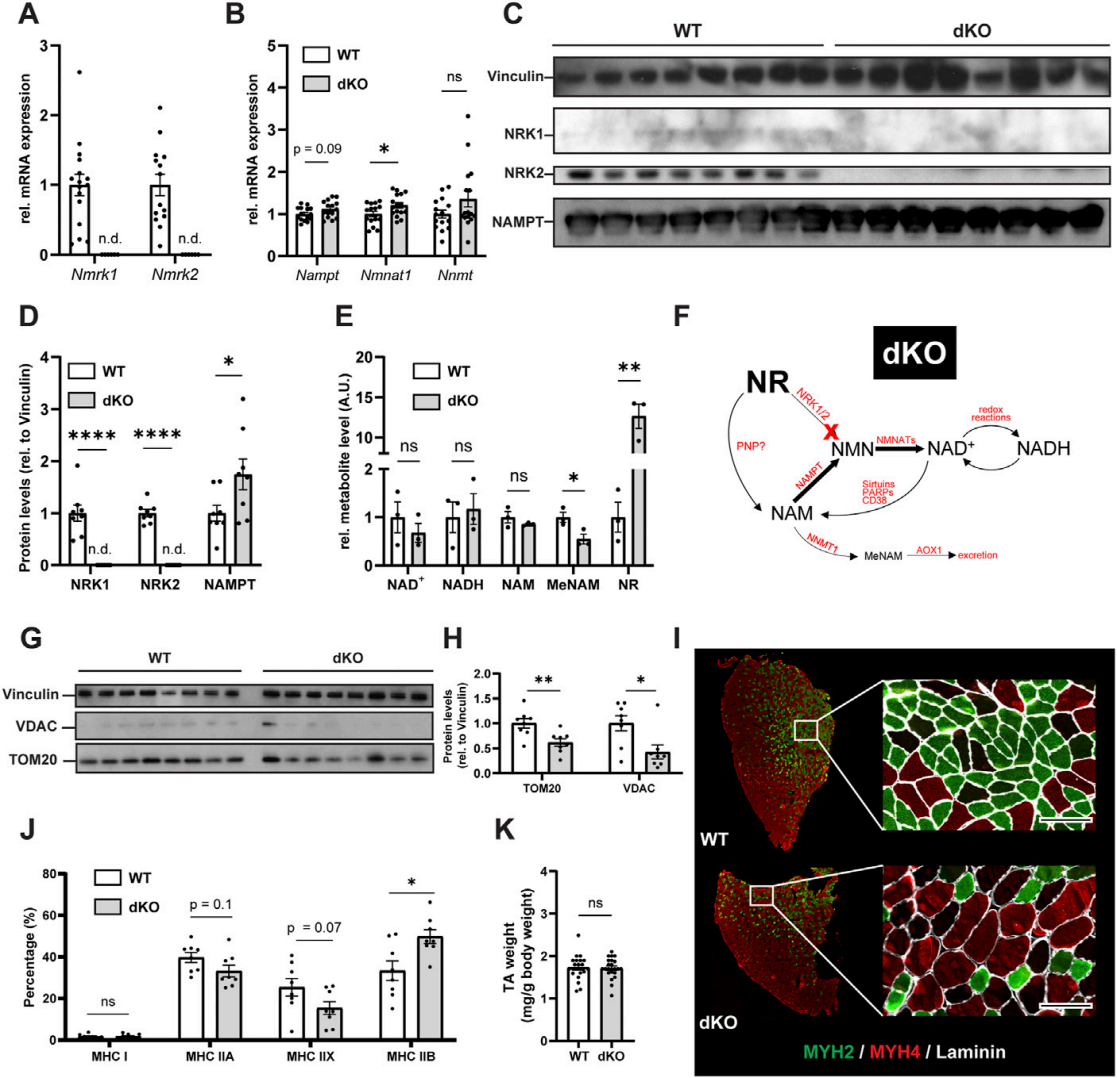
NRKdKO alters the NAD^+^ metabolome, impairs mitochondria and induces a shift to fast glycolytic fiber types. **(A,B)** mRNA expression levels of NR converting enzymes **(A)** and of the major skeletal muscle NAD^+^ biosynthesis (*Nampt*, *Nmnat1*) and NAM excretion (*Nnmt*) enzymes **(B)**. Gene expression of mRNA extracted from TA muscle relative to *Atp5b*, *Eif2a*, and *Psmb4* as housekeeping genes. n = 16. **(C,D)** Representative immunoblot images **(C)** and quantification **(D)** by densitometry analysis of NRK1/2 and NAMPT in WT in NRKdKO muscles. *n* = 8. **(E)** LC-MS/MS qualitative metabolomics targeted to NAD^+^, NADH, NAM, MeNAM, and NR in GC muscle. Shown are qualitatively measured metabolite levels relative to WT, peak area normalized to an internal standard and mg protein per sample. *n* = 3. **(F)** Proposed mechanism for altered NAD^+^ biosynthesis and utilization in NRKdKO muscle. NAM salvage to NAD^+^ is increased at the expense of NAM excretion. **(G,H)** Representative immunoblot images **(G)** and quantification **(H)** by densitometry analysis of VDAC and TOM20 in WT and NRKdKO muscles. n = 8. **(I,J)** Representative immunofluorescence images **(I)** and quantification **(J)** of fiber types in WT and NRKdKO TA muscle sections. Myh2 (Type IIA), green; Myh4 (Type IIB), red; laminin, gray. Scale bar, 100 µm *n* = 8. **(K)** TA wet weight from WT and NRKdKO mice. *n* = 16. Results shown are mean ± s. e.m. with *p < 0.05, **p < 0.01, ****p < 0.0001 *versus* WT, determined by unpaired Student’s t-test.

The final steps of NAD^+^ biosynthesis are mediated by two parallel branches that recycle NAM *via* a NAMPT-dependent salvage pathway or synthesize NAD^+^ *de novo* from NR *via* NRKs (Figure 1A). To better understand the role of these pathways in skeletal muscle, we first analyzed the skeletal muscle expression of *Nampt*, *Nmrk1* and *Nmrk2* (encoding NRKs) in mononucleated cells and myofibers using publicly available single-cell and single-nuclei RNAseq datasets covering both healthy and regenerating muscle. *Nampt* is broadly expressed, with high expression in all types of myofibers and lower but significant expression in most mononucleated cells such as myogenic progenitors, FAPs, tenocytes, endothelial cells, and immune cells (Figures 1B–D; Supplementary Figure S1A). In contrast, the expression of NRKs is much more restricted with *Nmrk1* having low basal expression in some mononucleated cells and *Nmrk2* having a high and selective expression in myofibers, with strong enrichment in type IIx fibers (Figures 1B–D; Supplementary Figure S1A). Consistent with this observation, the expression of *Nmrk2* was primarily detected in type IIx fibers of fast muscles, but not in slow muscles with low amounts of type IIx fibers (Supplementary Figures S1B,C), thus prompting us to study the role of NRKs in either fast or mixed muscles. The role of NRKs has been previously studied in mouse models of NRK1 or NRK2 single KO (sKO) (Ratajczak et al., 2016; Fletcher et al., 2017; Deloux et al., 2018). From the single cell profiling atlas, NRK2 is the most abundant isotype in skeletal muscle and seems the most relevant to study. Nevertheless, given the low expression of NRK1 levels in some mononucleated cells and the possibility that each NRK may compensate the loss of the other in single KO studies (Fletcher et al., 2017), we decided to study mice deficient for both NRK one and 2 (full body NRKdKO). The previous reports on the inability of NRKdKO cells to efficiently convert exogenous NR into NAD^+^ (Ratajczak et al., 2016; Fletcher et al., 2017) prompted us to analyze whether the endogenous muscle NAD^+^ metabolome is altered in the absence of both NRKs. As expected, expression of *Nmrk1* and *Nmrk2* mRNA was not detected in skeletal muscle of NRKdKO mice (Figure 2A). The expression of the two most highly expressed enzymes for NAD^+^ salvage in skeletal muscle, *Nampt* and *Nmnat1* (Fletcher et al., 2017), showed a mild increase in NRKdKO muscles (Figure 2B), a tendency that was further confirmed at the protein level (Figures 2C,D). As expected, neither NRK1 nor NRK2 were detected at the protein level in NRKdKO skeletal muscle (Figures 2C,D). NRK1/2 deletion did not induce transcriptional compensation through *Pnp* (Figure 1A), an alternative route of NR metabolism known from yeast (Bieganowski and Brenner, 2004) and possibly active in mammals too (Kulikova et al., 2019) (data not shown). In line with previous reports (Fletcher et al., 2017), NAD^+^ levels measured by LC-MS metabolomics were not altered in NRKdKO compared to WT GC muscle (Figure 2E), suggesting that the absence of NRKs might be compensated by alternative routes of NAD^+^ biosynthesis. Using LC-MS metabolomics, we demonstrated that NR is enriched more than 10-fold in NRKdKO muscle (Figure 2E), while methyl-nicotinamide (MeNAM) was lower in NRKdKO muscle (Figure 2E). The absence of NRKs therefore alters salvage of NAM into the NAD^+^ cycle at the expense of MeNAM, which is the NAD^+^ metabolite excreted in the urine (Pissios, 2017) and therefore spared in NRKdKO mice. Reduced MeNAM elimination from NRKdKO muscle cannot be explained by changes in expression of enzymes degrading NAM outside of the NAD^+^ pathway such as *Nnmt* (Figure 2B), but may be linked to increased NAM salvage (Figure 2F), given the increased expression of *Nmnat1* (Figure 2B) and NAMPT (Figures 2C,D). Thus, our data show that NR is an endogenous metabolite in muscle with an active bioconversion to NAD^+^ under normal homeostatic conditions, and that absence of NRKs is compensated by increased NAM salvage.

NAD^+^ metabolism plays a key role in maintaining mitochondrial fitness and homeostasis (Cantó et al., 2015). Absence of *Nmrk1 and Nmrk2* in skeletal muscle resulted in fewer mitochondria compared to WT muscles as indicated by reduced protein levels of TOM20 and VDAC (Figures 2G,H). Since mitochondrial density in skeletal muscle varies among fiber types and is reflective of the main energy substrate used by myofibers (Glancy and Balaban, 2011), we investigated whether the reduced mitochondrial content in NRKdKO muscles was associated with a fiber type shift. TA muscles from NRKdKO mice had more type IIB glycolytic fibers characterized by a lower mitochondrial content (Figures 2I,J). The increase in the number of type IIB fibers was paralleled by a decrease in the amount of type IIA oxidative and type IIX intermediate fibers (Figures 2I,J). In contrast, muscle mass did not vary between WT and NRKdKO mice (Figure 2K). Altogether, our data show that the deletion of NRK1/2 does not cause NAD^+^ deficiency in skeletal muscle but alters the turn-over of the NAD^+^ metabolome, leading to perturbed mitochondria content and reduced proportion of oxidative fibers.

### NRKdKO does not alter muscle wasting induced by denervation

**FIGURE 3:**
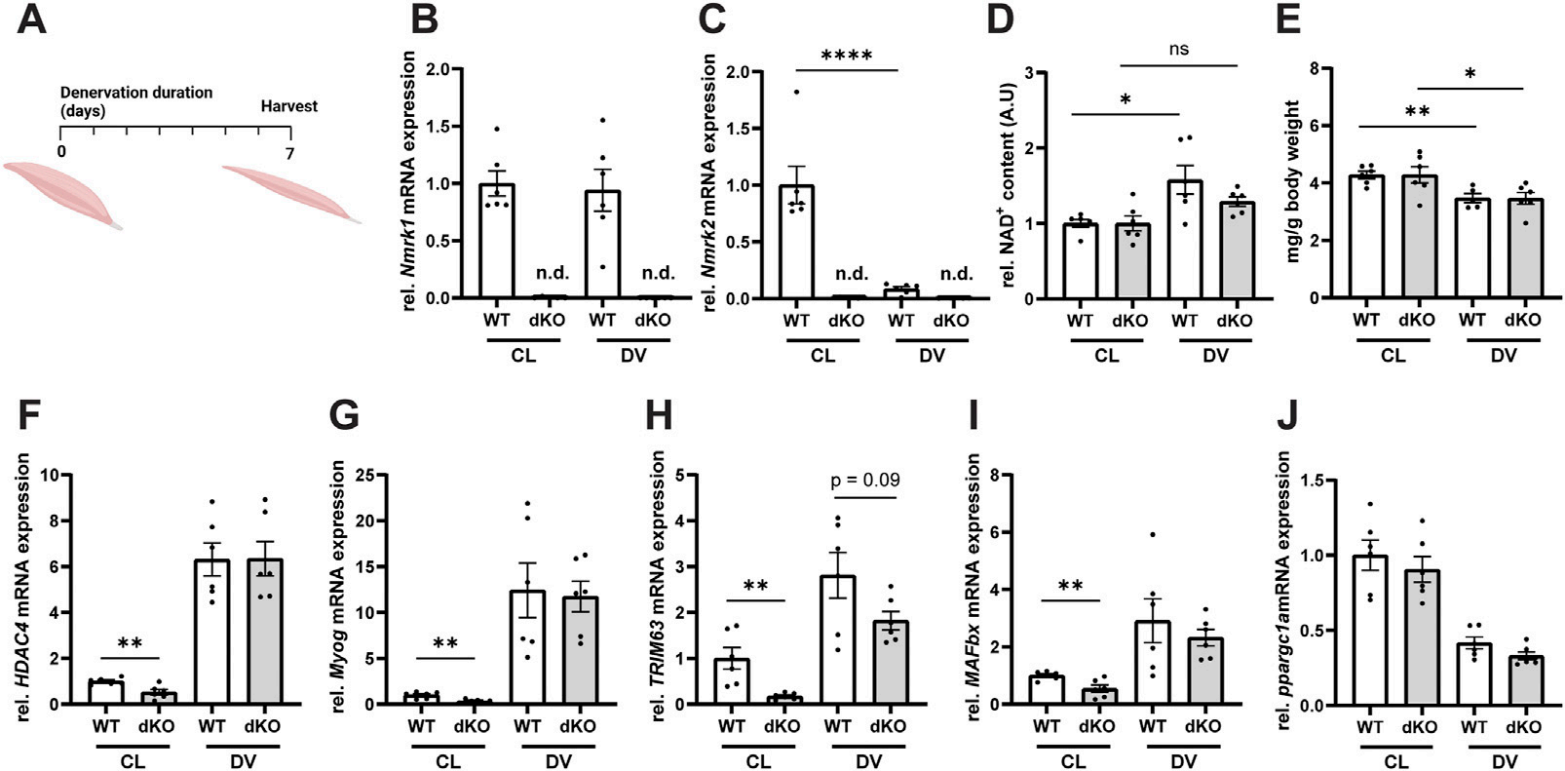
Loss of NRK1 and NRK2 does not affect the transcriptional regulation of denervation-induced muscle atrophy. **(A)** Timeline of the denervation experimental procedure. Mice were subjected to unilateral resection of the sciatic nerve and tissues were collected 1 week after denervation. **(B,C)** mRNA expression levels of *Nmrk1* **(B)** and Nmrk2 **(C)**. Gene expression of mRNA extracted from TA muscle measured by qPCR relative to *bActin* as housekeeping gene. **(D)** Relative NAD^+^ content of GC muscle quantified by NAD^+^ cycling assay. **(E)** Weight of GC muscle normalized to whole body weight. **(F–J)** mRNA expression levels of genes regulated by denervation. Results shown are mean ± s. e.m. with *p < 0.05, **p < 0.01, ****p < 0.0001 *versus* WT, determined by unpaired Student’s t-test, n = 6. CL, contralateral and DV, denervated. Created with BioRender.com.

Given our observation that deletion of NRK1/2 alters NAD^+^ utilization in healthy skeletal muscle (Figures 2E,F), we reasoned that pathological conditions could further alter the balance of NAD^+^ biosynthesis routes and lead to more severe defects on NAD^+^ metabolism and muscle physiology. To investigate this possibility, we first induced muscle wasting by performing unilateral resection of the sciatic nerve in NRKdKO and WT control mice (Figure 3A). In WT mice, *Nmrk2* expression was strongly downregulated during denervation while *Nmrk1* remained expressed at normal levels (Figures 3B,C), thus highlighting a possible role of NRK2 in regulating skeletal muscle size and homeostasis. As expected, *Nmrk1/2* expression was not detected in NRKdKO muscles (Figures 3B,C). Muscle NAD^+^ levels increased in response to denervation-induced muscle atrophy in WT mice, but NRKdKO mice did not elevate NAD^+^ levels after denervation, indicating that NRKs become limiting to adapt NAD^+^ levels to the absence of innervation and contraction (Figure 3D). Despite this difference in NAD^+^ levels, denervation induced similar muscle wasting in WT and NRKdKO mice, with both groups losing 20% of muscle mass compared to the contralateral innervated muscle (Figure 3E). Gene expression analysis confirmed that the *Hdac4*/*Myogenin* transcriptional network activated upon denervation (Moresi et al., 2010) was induced at similar levels in both genotypes (Figures 3F,G). In addition, downstream atrogenes like *TRIM63* and *MAFbx*, which encode the E3 ligases MuRF1 and Atrogin1 mediating muscle protein breakdown, were induced normally in the denervated muscles of NRKdKO mice (Figures 3H,I). Down-regulation of *Ppargc1a* in response to denervation was also unaffected in NRKdKO muscle (Figure 3J). While denervation induced a similar response in WT and NRKdKO mice, basal expression levels of most atrogenes in NRKdKO mice were lower compared to WT mice (Figures 3H,I), suggesting that NRK1/2 deletion could slightly modify the anabolic/catabolic balance controlling fiber size but at levels that are not sufficient to regulate muscle mass (Figure 2K). Altogether, our results demonstrate that NRKs might be subtle regulators of skeletal muscle catabolism in homeostatic conditions but that modulation of NAD^+^ metabolism by NRK1/2 does not impact the loss of muscle mass during denervation-induced atrophy.

### NRKdKO MuSCs hyperproliferate at the expense of differentiation

**FIGURE 4:**
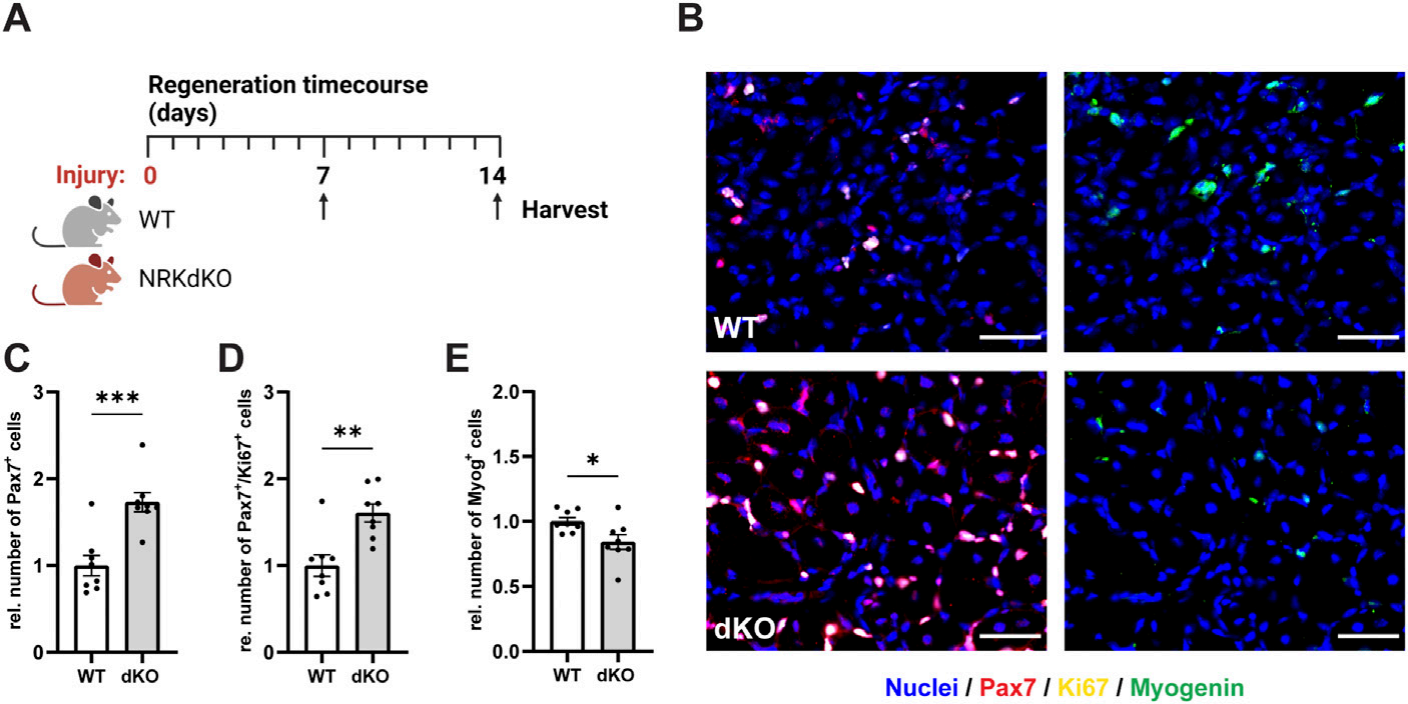
NRKdKO perturbs MuSC proliferation and differentiation during muscle regeneration. **(A)** Experimental design of the regeneration time course after cardiotoxin (CTX)-induced muscle injury. **(B–E)** Representative histological images **(B)** and quantification of total MuSCs **(C)**, proliferating MuSCs expressing Ki67 **(D)**, and Myogenin-positive cells **(E)** at 7 dpi. Pax7, red; Ki67, yellow; Myogenin, green; nuclei (Hoechst), blue. Scale bars, 50 µm. Results shown are mean ± s. e.m. with *p < 0.05, **p < 0.01, ***p < 0.001 *versus* WT, determined by unpaired Student’s t-test, *n* = 8. Created with BioRender.com.

We then asked whether NRK1/2 could play a more prominent role in conditions where muscle fibers are damaged and require tissue repair mechanisms and MuSC activity. We thus challenged the hindlimb muscles of WT and NRKdKO mice with an intramuscular injury of cardiotoxin (CTX) and analyzed MuSCs 7 days post-injury (dpi), when myogenic progenitors withdraw from cell cycle, differentiate and fuse to damaged or new myofibers (Figure 4A) (Bentzinger et al., 2013). Absence of *Nmrk1* and *Nmrk2* resulted in a hyperproliferative phenotype at 7 dpi characterized by a higher number of Pax7-positive cells and by more Pax7/Ki67 double positive proliferating myogenic progenitors (Figures 4B–D). The persistence of MuSCs in the proliferative phase was not due to a prolonged inflammatory response as both the expression of macrophage markers (Supplementary Figures S2A–C) and of the cytokines TNFα, IL-1b, and IL-6 (Supplementary Figures S2D–F) were unchanged between WT and NRKdKO mice at 7dpi. Importantly, progenitor differentiation was delayed as the number of Myogenin-positive cells that commit to terminal differentiation for tissue repair was reduced (Figures 4B,E). These data demonstrate that NRKdKO MuSCs have altered myogenic dynamics during tissue repair.

### NRKdKO impairs NAD^+^ recovery and alters mitochondrial adaptation during muscle regeneration

**FIGURE 5:**
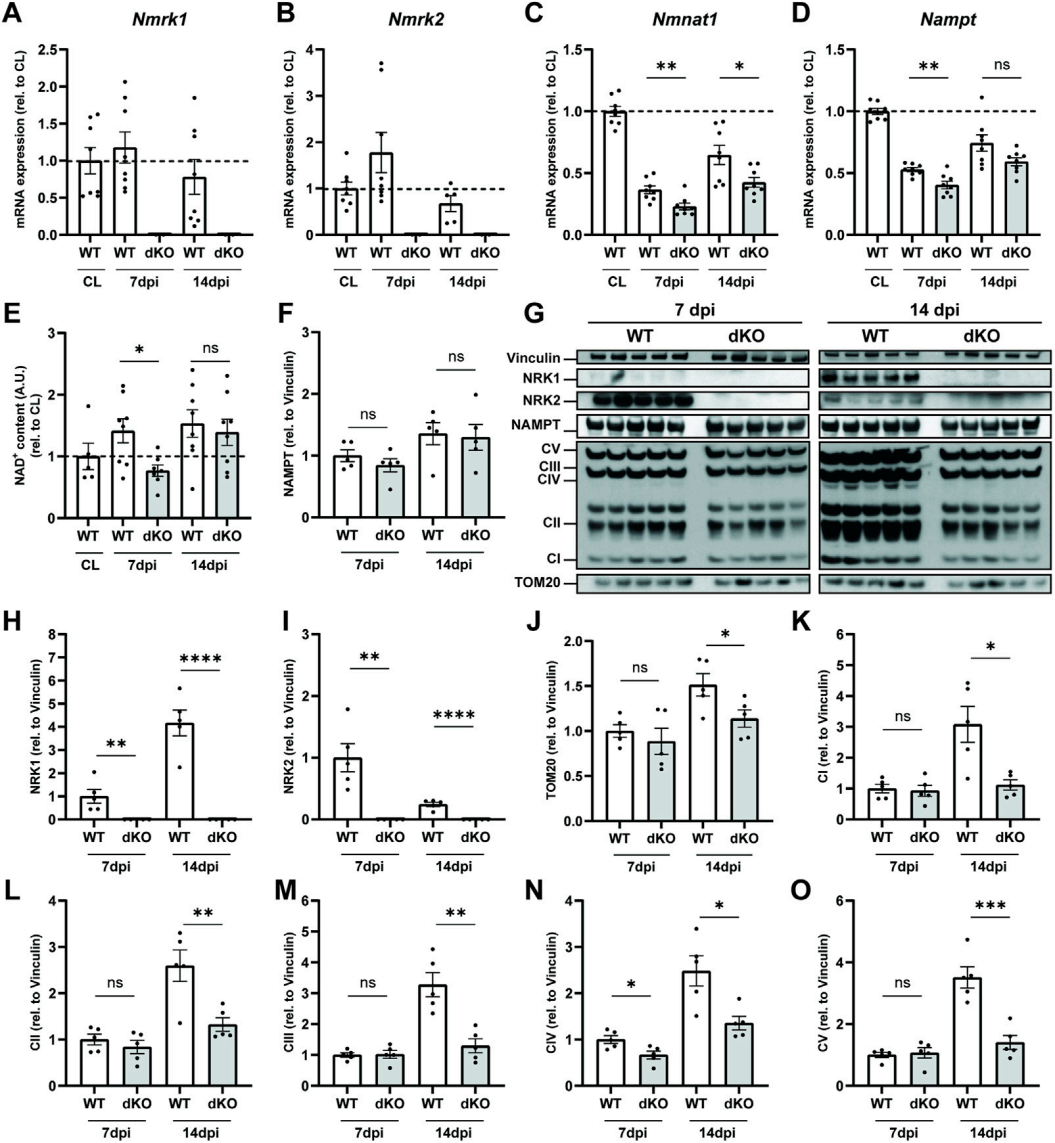
NRKdKO delays regeneration of NAD^+^ levels and mitochondrial bioenergetics after muscle injury. Muscle regeneration was performed as represented in Figure 4. **(A–D)** *Nmrk1* **(A)**, *Nmrk2* **(B)**, *Nmnat1* **(C)** and *Nampt* **(D)** mRNA expression at 7 and 14 dpi measured by qPCR relative to *Atp5b*, *Eif2a*, and *Psmb4* as housekeeping genes, *n* = 8. **(E)** Relative NAD^+^ levels in TA muscle measured by cycling assay. **(F–O)** Representative immunoblot images and quantification by densitometry analysis of NAD^+^ salvage enzymes **(F–I)** and mitochondrial markers **(J–O)**, *n* = 5. Results shown are mean ± s. e.m. with *p < 0.05, **p < 0.01, ***p < 0.001, ****p < 0.0001 *versus* WT determined by unpaired Student’s t-test. CL contralateral uninjured muscle.

In parallel to the MuSC phenotype, we investigated how the loss of NRKs impacts the regulation of NAD^+^ levels and mitochondrial metabolism. In WT mice, muscle injury did not influence expression of *Nmrk1* (Figure 5A) and transiently increased *Nmrk2* expression at seven dpi (Figure 5B). The peak in *Nmrk2* expression at 7 dpi coincides with early fiber formation and is consistent with data previously reported in C2C12 cells (Fletcher et al., 2017), overall supporting a role for NRK2 during progenitor differentiation. The recovery of NAD^+^ biosynthesis/salvage enzymes was slowed down in regenerating NRKdKO muscle as re-expression of both *Nampt* and *Nmnat1* during muscle repair was delayed (Figures 5C,D). Functionally, the recovery of NAD^+^ was also slower in NRKdKO muscles as NAD^+^ levels were lower at seven dpi but then fully recovered at 14 dpi once regeneration has completed (Figure 5E). Similarly, NAMPT protein levels tended to be lower at 7 dpi before recovering at 14 dpi (Figures 5F,G).

At the protein level, we observed an increase in NRK1 from 7 to 14 dpi as myofibers matured (Figures 5G,H). In contrast, NRK2 protein levels peaked at seven dpi before going back down at 14 dpi (Figures 5G,I), thus validating prior results obtained by us and others (Fletcher et al., 2017) at the molecular level and implying a role for NRK2 during progenitor differentiation. Regenerating muscles underwent substantial mitochondrial remodeling with a strong increase in mitochondrial biogenesis from 7 to 14 dpi as newly generated myofibers matured (Figures 5G,J). However, absence of NRK1/2 very consistently impaired the mitochondrial adaptation of all respiratory chain complexes at 14 dpi during terminal metabolic maturation of newly formed and repaired myofibers (Figures 5G,K–O). Thus, regeneration amplified the mitochondrial phenotype observed in NRKdKO uninjured muscles (Figures 2G,H). In summary, deletion of NRK1/2 delays recovery of the NAD^+^ pool after muscle injury by preventing the conversion of NR into NAD^+^ in regenerating myofibers which have not yet turned on NAM salvage. Moreover, the absence of NRK1/2 alters the metabolic adaptation of newly formed myofibers, demonstrating that NR-derived NAD^+^ becomes limiting in newly formed muscle fibers during muscle regeneration.

### Delayed maturation of newly formed fibers in NRKdKO muscle

**FIGURE 6:**
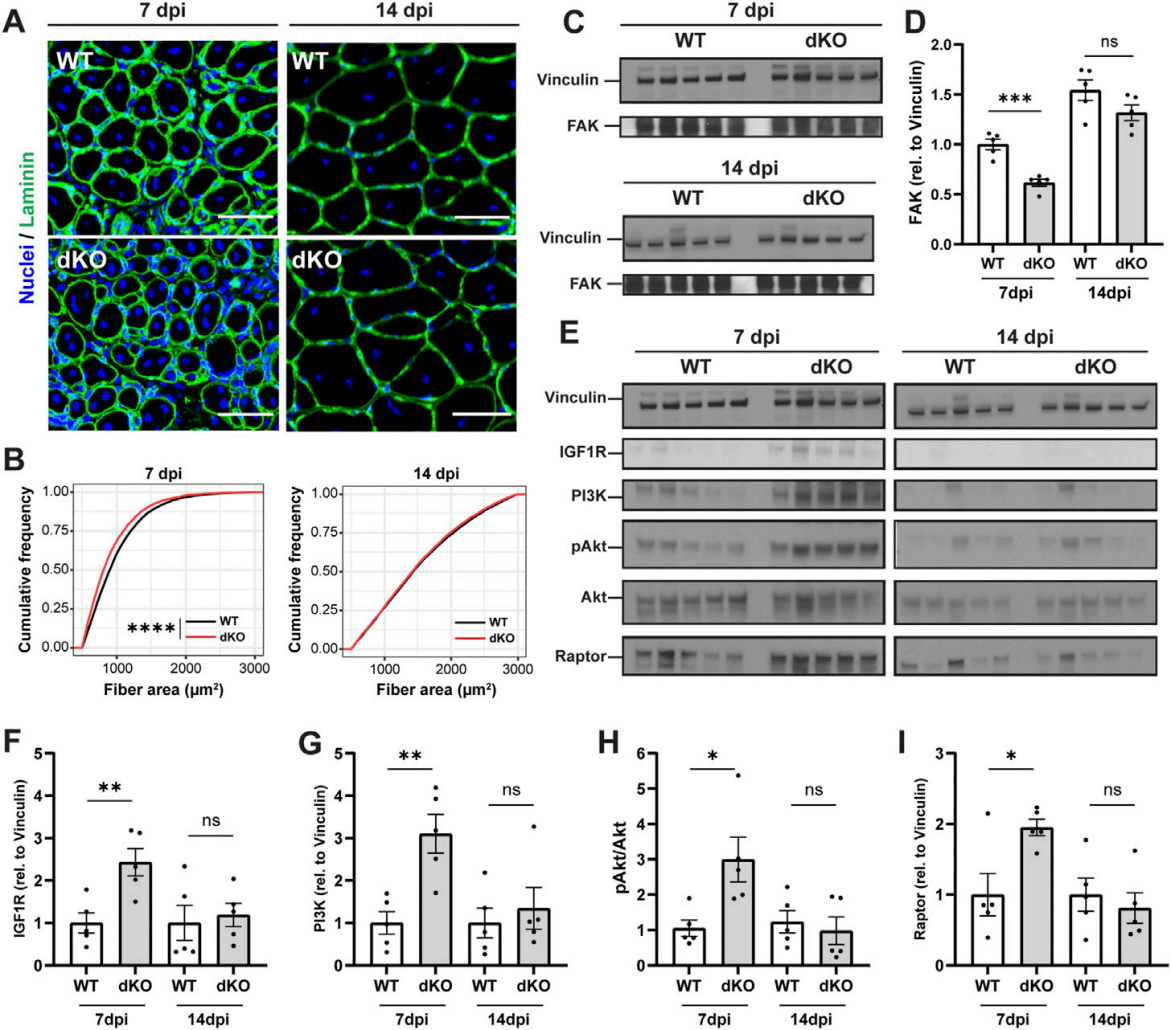
NRKdKO delays the regeneration of injured myofibers which recover through compensatory IGF1/AKT signaling. Mice were subjected to unilateral CTX injection into hindlimb muscle, tissues were collected 7 and 14 dpi. **(A)** Representative images of TA muscle sections stained with laminin (green) and Hoechst for nuclei (blue) at 7 dpi and 14 dpi. Scale bar, 50 µm *n* = 8. **(B)** Cumulative distribution of cross-sectional area of regenerating fibers with centralized nuclei at 7 dpi and 14 dpi. **(C,D)** Representative immunoblot images **(C)** and quantification **(D)** by densitometry analysis of Focal Adhesion Kinase (FAK) in WT in NRKdKO muscles, *n* = 5. **(E–I)** Representative immunoblot images **(E)** and quantification **(F–I)** by densitometry analysis of the AKT pathway in WT in NRKdKO muscles, *n* = 5. Results shown are mean ± s. e.m. with *p < 0.05, **p < 0.01, ***p < 0.001, ****p < 0.0001 *versus* WT, determined by unpaired Student’s t-test **(D,F–I)** or **(B)** Kolmogorov-Smirnov test.

To further examine how the stem cell and metabolic phenotypes of NRKdKO mice impact the maturation of muscle fibers during the time course of regeneration, we investigated myofiber size and extracellular matrix (ECM) remodeling of regenerating muscles at 7 and 14 dpi. NRKdKO mice had a transient reduction of the cross-sectional area of newly formed myofibers with centralized nuclei at seven dpi (Figures 6A,B). This myofiber phenotype paralleled extracellular matrix remodeling defects with thinner ECM and laminin and more rounded fibers at 7dpi in NRKdKO muscle (Figure 6A). The ECM is heavily remodeled during tissue repair and is both a structural and a signaling cue that facilitates myofiber maturation (Bentzinger et al., 2013). We confirmed that other components of the ECM are probably abnormally remodeled at 7dpi as NRKdKO decreased the expression of the Focal Adhesion Kinase (FAK) (Figures 6C,D), an intracellular mediator of ECM/integrin signaling (Moreno-Layseca and Streuli, 2014; Sulzmaier et al., 2014; Lukjanenko et al., 2016). Surprisingly, despite the clear mitochondrial defect at 14 dpi (Figures 5G–O), the size of regenerating NRKdKO myofiber had recovered at 14 dpi (Figures 6A,B). This prompted us to investigate whether the metabolic defect was partially compensated by other molecular mechanisms. Muscle fiber size is positively regulated by the IGFR-Akt-mTOR pathway during myofiber maturation (Schiaffino et al., 2013). When interrogating this pathway, we observed a strong, transient upregulation of IGFR, PI3K, AKT, and the downstream effector Raptor at 7dpi in NRKdKO mice (Figures 6E–I). This anabolic compensation was transient as it had recovered at 14 dpi (Figure 6E). Overall, these data demonstrate for the first time that the absence of *Nmrk1* and *Nmrk2* transiently delays skeletal muscle regeneration, and that this phenotype is compensated by an adaptive activation of anabolic signaling to keep up with the regenerative demand and meet homeostatic requirements.

## Discussion

Muscle NAD^+^ biosynthesis relies mainly on NAM salvage by NAMPT, which contributes up to 85% of muscle NAD^+^ synthesis in healthy muscle (Frederick et al., 2015; Frederick et al., 2016; Fletcher et al., 2017). Nevertheless, the muscle-specific expression of a second NRK, NRK2, strongly suggests that NR plays a specific role as an NAD^+^ precursor in skeletal muscle (Fletcher and Lavery, 2018). Despite the overlapping function of the NRK isozymes in phosphorylating NR, available loss of function models mainly focus on NRK1 or NRK2 single deletions in mice and only a limited amount of data describing the phenotype of NRKdKO muscle has been presented up to date. It was reported that NRK1 and NRK2 sKO and NRKdKO mice do not exhibit significant differences in their muscle tissue NAD^+^ levels neither at baseline nor during aging (Ratajczak et al., 2016; Fletcher et al., 2017; Deloux et al., 2018). Our results corroborate these findings and bring fundamental new insights using metabolomics by demonstrating that an NRK-mediated metabolization of NR is active in healthy muscle, since the absence of NRKs leads to a significant accumulation of NR. This important result highlights the rate-limiting nature of NRKs for the efficient utilization of NR as a muscle NAD^+^ precursor, as previously shown in primary cells isolated from NRK KO mice (Ratajczak et al., 2016).

In our study, NAM levels did not differ between the genotypes likely because of its high turn-over, but we observed significantly lower levels of the NAM catabolite MeNAM. This observation could be linked to a generally lower NAD^+^ turnover in NRKdKO muscle. However, increased levels of NAMPT in NRKdKO muscle rather suggests that NAM recycling is favored over NAM excretion in the absence of NRKs.

Despite the observation that muscle NAD^+^ levels remain unchanged in NRKdKO mice, deletion of NRK1/2 results in a significant reduction of mitochondrial content under homeostatic conditions. One hypothesis for the lower mitochondrial content lies into the distribution of mitochondria within skeletal muscle fibers. Mitochondrial mass in oxidative fibers is two to three times that of fast-twitch glycolytic fibers (Lin et al., 2002). Since loss of NRK1/2 triggers a fiber type shift to glycolytic fibers, one explanation for the reduced mitochondrial volume in NRKdKO mice could thus be the shift to glycolytic fibers at the expense of oxidative fibers. It is likely that the fiber type shift of NRKdKO mice is primarily driven by NRK2 since it is more highly expressed in muscle than NRK1 and NRK2 sKO mice also present a similar fiber type switch (Deloux et al., 2018). Our profiling of single cell/myonuclei further corroborated this observation by demonstrating that NRK1 is not expressed in fibers while NRK2 has strong fiber-type specific expression with predominant expression in type IIx fibers, thus providing a direct cellular explanation to the IIx to IIB fiber type switch detected in NRKdKO. Studies using NAMPT muscle-specific KO mice or pharmacological inhibition of NAMPT *via* FK866 showed that severe NAD^+^ depletion in NAMPT-deficient cells compromises mitochondrial bioenergetics and exacerbates anaerobic glucose metabolism (Frederick et al., 2016; Tan et al., 2019). Interestingly, the mitochondrial phenotype of NRKdKO muscle occurs with metabolomic signs of a perturbed NAD^+^ utilization but in the absence of detectable changes of muscle NAD^+^ levels, suggesting that mitochondria are sensitive to subtle changes of NAD^+^ catabolism. The fact that NRKdKO impairs mitochondrial content shows for the first time that NRKs and the endogenous NR path to NAD^+^ plays an important role in muscle oxidative metabolism *in vivo.*

Due to its muscle-specific expression, the role of NRK2 has been previously studied in different *in vitro* and *in vivo* models (Li et al., 1999; Goody et al., 2010; Fletcher et al., 2017; Deloux et al., 2018). In C2C12 mouse myoblasts, NRK2 was first described as the muscle integrin binding protein (MIBP) and reported to localize to the plasma membrane, binding the cytosolic tail of the laminin receptor integrin α7β1 (Li et al., 1999). Zebrafish muscle development strongly depends on NRK2b (Goody et al., 2010), and even though mice deficient of NRK1 and/or NRK2 develop grossly normal, aged NRK2 sKO mice demonstrated a maladaptive response to exercise (Deloux et al., 2018). Additionally, *Nmrk2* has been observed to be upregulated in response to energy stress in the failing heart of a mouse model of dilated cardiomyopathy as well as in human heart failure (Diguet et al., 2018). Interestingly, *Nmrk2* appears to be a stress-responsive transcript also in non-muscle tissues, as observed in dorsal root ganglions after sciatic nerve resection (Sasaki et al., 2006), suggesting that roles of NRK2 in mammals could relate to adaptations to physiological challenges.

Despite the rather mild phenotypes of NRKdKO muscle under normal homeostasis, our results demonstrate a distinct role of NRKs in some situations where muscle plasticity is challenged. NRK1/2 deletion did not influence muscle atrophy induced by denervation where the cytoplasmic muscle contractile apparatus is degraded by E3-ligase mediated proteasomal degradation in the absence of neuromuscular stimulation (Beehler et al., 2006; Moresi et al., 2010), but without damage to myofiber membranes. This could be linked to the fact that NRK2 expression is maintained *via* innervation and excitation/contraction coupling but actually rapidly declines in muscle after denervation.

In contrast, the severe damage of muscle fibers after muscle injury leads to a transient decline in NAD^+^ levels early after injury that coincides with the downregulation of NAMPT for NAD^+^ recycling *via* the salvage pathway. Interestingly, NRKdKO muscle has delayed recovery of NAD^+^ during regeneration, demonstrating that NRKs becoming rate-limiting for the salvage of NAD^+^ when NAM is no longer dominant. The expression of NRKs is maintained or even slightly elevated to maintain NAD^+^ biosynthesis capacity during the early phase of muscle regeneration, as also previously reported for NRK2 where *Nmrk2* is upregulated in mouse muscle in response to freeze injury (Sasaki et al., 2006) and during *in vitro* myoblast differentiation (Aguilar et al., 2015). In line with this observation, absence of NRK1/2 decreased the number of Myogenin-positive progenitors during regeneration and affected their differentiation capacity. This was paralleled by a hyperproliferative phenotype of MuSCs, demonstrating that NR metabolization through NRKs is not limiting for MuSC activation and cell cycle entry but is required for efficient differentiation of cycling MuSCs. Interestingly, the MuSC and NAD^+^ defects induced a transient delay of regeneration and of myofiber maturation. Single cell profiling revealed that NRK2 expression is restricted to fibers while low levels of NRK1 is detected in mononucleated cells, including FAPs and myogenic cells during regeneration. While it is important to realize that single cell RNAseq may lack the sensitivity to detect the expression of transcripts with low expression given the limited sequencing depth and may bias the exploration of which niche cells drive regenerative phenotypes, these results suggest that MuSC/myogenic phenotypes are primarily mediated by NRK1 while phenotypes of NAD^+^ and metabolic recovery myofiber are largely governed by NRK2. Regenerating fibers were smaller and failed to induce the expression of the different complexes of the mitochondrial electron transport chain in NRKdKO muscle, demonstrating that the perturbed NAD^+^ utilization impairs the metabolic maturation of newly formed myofibers during tissue repair. Despite these transient delays, regeneration could actually proceed normally at the later stages of tissue repair as a robust IGF/Akt anabolic compensation was induced in NRKdKO regenerating muscle to allow tissue repair to complete. This adaption is an amplification of the Akt/mTOR anabolic signaling also at play at 7dpi in WT regenerating muscle to sustain the growth of newly formed or repairing fibers (Fletcher et al., 2017), and could be triggered by reduced inhibition of Sirt6 *via* low NAD^+^ in NRKdKO myofibers as Sirt6 inhibition has recently been shown to activate IGF-Akt-mTOR signaling in muscle *via* c-jun activation (Mishra et al., 2022).

The recovery of muscle architecture after regeneration also requires efficient re-organization of the extracellular matrix composing the basal lamina, a process that is important for fiber maturation and reacquisition of contractile and metabolic characteristics of each newly-formed myofiber (Pette and Vrbová, 1985; Pette and Staron, 2001; Schiaffino and Serrano, 2002). Based on their findings during zebrafish muscle development, Goody and Henry (Goody et al, 2010; Goody and Henry, 2018) proposed a model in which membrane-located NRK2 constantly generates a membrane-proximal pool of NAD^+^. In this model, muscle damage leads to NAD^+^ leakage into the extracellular space and might also stimulate the active transport of NAD^+^ across the sarcolemma of intact fibers (Zolkiewska, 2005). Extracellular ADP-ribosyltransferases (i.e. ART1) are then able to utilize local NAD^+^ for ADP-ribosylation of integrin α7β1, which enhances the affinity for integrin-laminin binding (Zhao et al., 2005). This contributes to increased laminin organization and further enables the intracellular localization of paxillin to cell-matrix adhesion complexes, finally resulting in efficient adhesion of regenerated muscle fibers (Goody et al., 2010). Consistent with these findings, we observed transient impaired ECM remodeling during muscle regeneration of NRKdKO mice with altered laminin remodeling and impaired levels of FAK, a intracellular mediator of ECM signaling (Moreno-Layseca and Streuli, 2014; Sulzmaier et al., 2014; Lukjanenko et al., 2016). Thus, our results suggest that the regulation of ECM by NRK2 *via* NAD + compartimentalization is also at play in mammalian skeletal muscle during regeneration.

Our data from NRKdKO mice provide the first evidence that NRK1 and two become the critical NAD^+^ producers after muscle injury and affect MuSCs and myofiber repair. It is possible that NRKs may regulate some aspects of tissue repair independent of the effects on NAD^+^, notably *via* their known role in the regulation of the extra-cellular matrix or phosphorylation of other substrates (Fletcher and Lavery, 2018). However, the altered levels of NAD^+^ in regenerating NRKdKO muscle and previous reports showing that exogenous NR restores NAD^+^ levels in aged and dystrophic muscle to boost MuSCs and regeneration (Ryu et al., 2016; Zhang et al., 2016) suggest that the regulation of NAD^+^ can mediate the biological effects of NRKs. Building on the model of Goody and Henry (Goody et al, 2010; Goody and Henry, 2018), our results support a model where NRKs may actually influence both the local NAD^+^ pool at the membrane for extracellular matrix remodeling and mechano-transduction to support MuSCs and newly formed fibers, while concomitantly regulating the cytoplasmic and mitochondrial NAD^+^ pool to regulate myogenic differentiation, myofiber maturation and recovery of the mitochondrial architecture in newly formed fibers.

In conclusion, we demonstrate that in healthy conditions, NRKs regulate an endogenous conversion of NR to NAD^+^ that has a minor contribution to the salvage of NAM but is sufficient to regulate mitochondrial content and fiber type specification. Transcriptional regulation of atrophy related signaling is not perturbed in NRKdKO muscle. However, in the context of muscle damage, our results demonstrate for the first time that NRK1/2 become the limiting NAD^+^ producing enzymes and are required for efficient MuSC differentiation and fiber maturation during regeneration. Our data overall support a key role for NRKs in skeletal muscle regeneration and highlight their requirement to maintain MuSC, mitochondria, and NAD^+^ homeostasis in skeletal muscle.

## Data Availability

The raw data supporting the conclusion of this article will be made available by the authors, without undue reservation.

## Supplementary material

Supplementary Figure S1 Expression of *Nampt*, *Nmrk1*, and *Nmrk2* in single-nuclei RNA-sequencing dataset of 5-month-old WT mice. **(A–C)** UMAP **(A)** and violin plots **(B,C)** of *tibialis anterior* **(B)** and *soleus* **(C)** muscles showing gene expression for myonuclear populations of *Nampt*, *Nmrk1*, and *Nmrk2*. The y-axis shows expression level as probability distribution across clusters. TA muscle samples from one mouse and pooled soleus samples from 4 mice were analyzed from Petrany et al. (38).

Supplementary Figure S2 CTX-induced immune response is not altered in NRKdKO muscle. mRNA expression of cellular surface markers of pro-inflammatory macrophages. **(A)** *F4/80* and **(B)** *CD11b*, and anti-inflammatory macrophages **(C)** *Msr1*. mRNA expression of cytokines **(D)** *Tnfa*, **(E)** *IL-1b*, and **(F)** *IL-6*. Gene expression of mRNA extracted from TA muscle measured by qPCR relative to *Atp5b*, *Eif2a*, and *Psmb4* as housekeeping genes. Results shown are mean ±- s.e.m. with **p* < 0.05, ***p* < 0.01 WT, determined by unpaired Student's t-test, *n*=6-8.
