# Supplementary material for: Nicotinamide riboside kinases regulate skeletal muscle fiber-type specification and are rate-limiting for metabolic adaptations during regeneration
Source: Front Cell Dev Biol. 2022 Nov 9;10:1049653. doi: 10.3389/fcell.2022.1049653 (PMC9682158; doi:10.3389/fcell.2022.1049653)
Supplement: Supplementary file 1 [file Image1.pdf]

Supplementary Material

1.1 Supplementary Figures

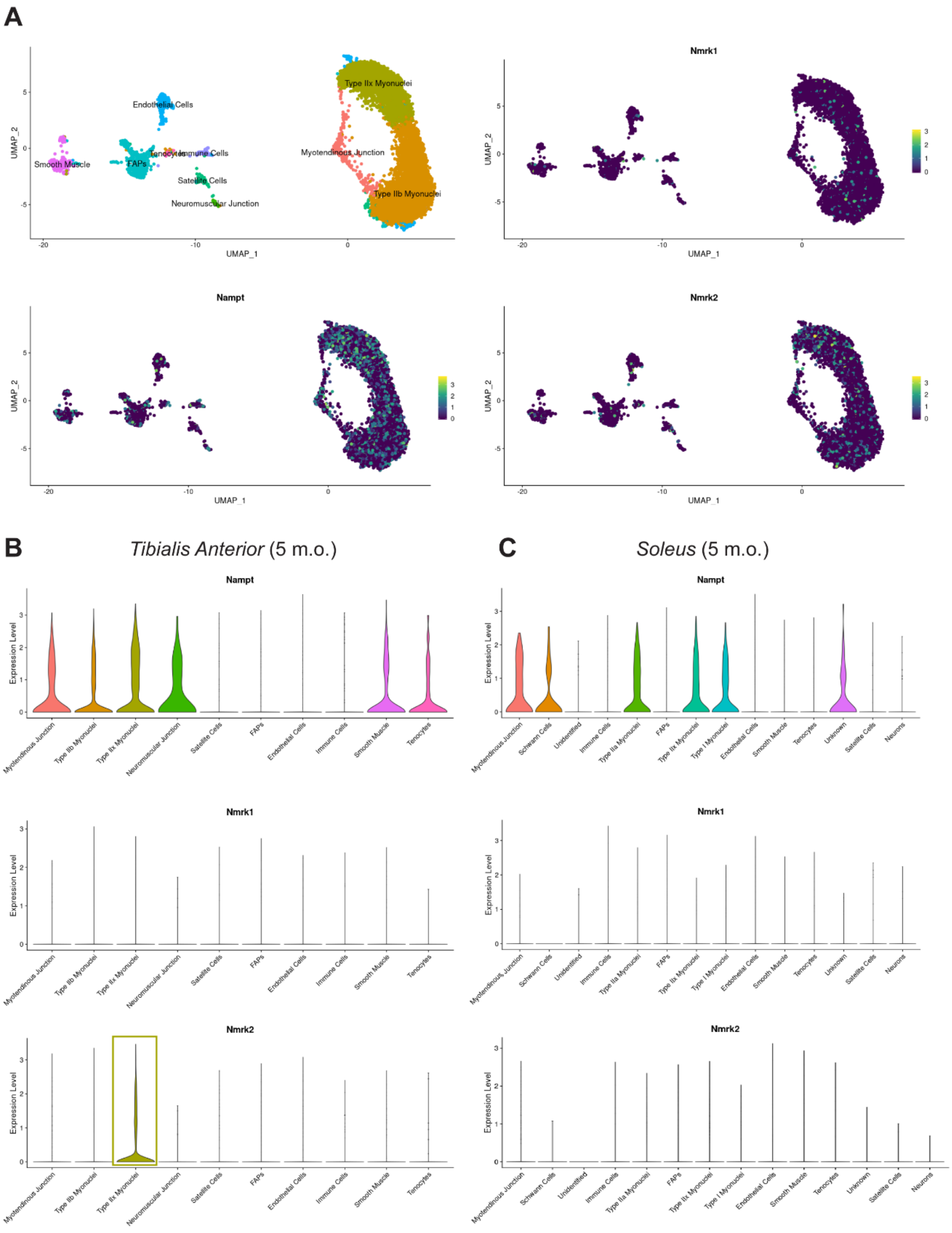

Supplementary Figure 1: Expression of *Nampt*, *Nmrk1*, and *Nmrk2* in single-nuclei RNA-sequencing dataset of 5-month-old (m.o.) WT mice. (**A-C**) UMAP (**A**) and violin plots (**B**,**C**) of *tibialis anterior* (**B**) and *soleus* (**C**) muscles showing gene expression for myonuclear populations of *Nampt*, *Nmrk1*, and *Nmrk2*. The y-axis shows expression level as probability distribution across clusters. TA muscle samples from one mouse and pooled soleus samples from 4 mice were analyzed from Petrany et al. (38).

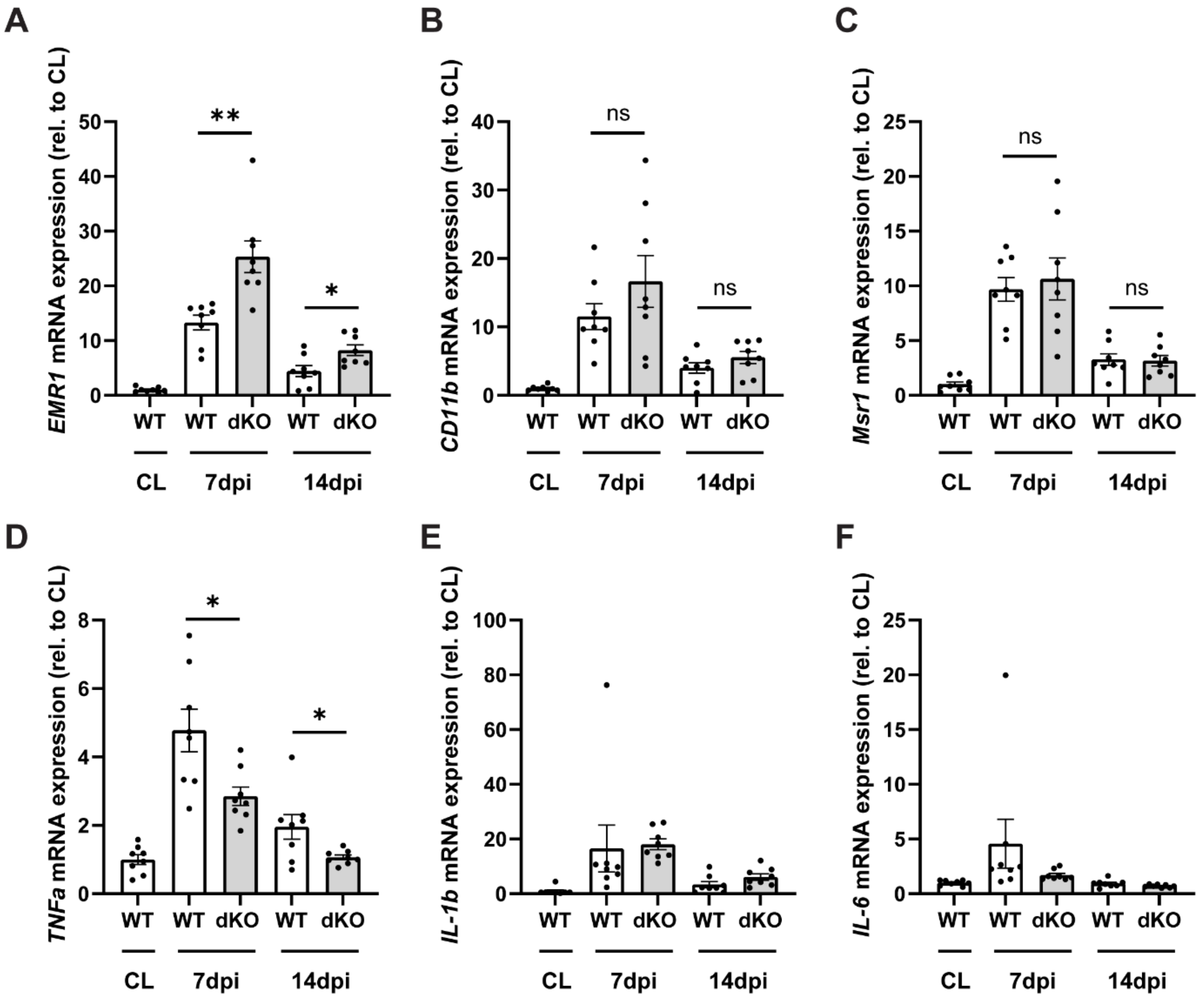

Supplementary Figure 2: CTX-induced immune response is not altered in NRKdKO muscle. mRNA expression of cellular surface markers of pro-inflammatory macrophages. (**A**) *F4/80* and (**B**) *CD11b*, and anti-inflammatory macrophages (**C**) *Msr1*. mRNA expression of cytokines (**D**) *Tnfa*, (**E**) *IL-1b*, and (**F**) *IL-6*. Gene expression of mRNA extracted from TA muscle measured by qPCR relative to *Atp5b*, *Eif2a*, and *Psmb4* as housekeeping genes. Results shown are mean ±- s.e.m. with *p < 0.05, **p < 0.01 WT, determined by unpaired Student's t-test, n=6-8.
